# Abnormal levels of expression of microRNAs in peripheral blood of patients with traumatic brain injury are induced by microglial activation and correlated with severity of injury

**DOI:** 10.1186/s40001-024-01790-y

**Published:** 2024-03-20

**Authors:** Shuo Feng, Zhangying Wu, Xianping Zheng, Zhiwei Shao, Qiang Lin, Shoutian Sun

**Affiliations:** 1 Department of Neurosurgery, Qingdao Huangdao District People’s Hospital, Qingdao, 266400 China; 2 Department of Cardiology, Qingdao Huangdao District People’s Hospital, Qingdao, 266400 China; 3 Intensive Care Unit, Zibo Central Hospital, Zibo, 255024 China; 4 Intensive Care Unit, Qingdao Huangdao District People’s Hospital, Qingdao, 266400 China; 5 Department of Neurosurgery, The Affiliated Hospital of Qingdao University, Qingdao, 266000 China; 6 Department of Emergency, Zibo Central Hospital, No. 54 Gongqingtuan Road, Zhangdian District, Zibo, 255024 China

**Keywords:** Traumatic brain injury, miRNAs, Microglia, Glasgow Coma Scale, Biomarker, ROC curve

## Abstract

**Background:**

Microglia play a crucial role in regulating the progression of traumatic brain injury (TBI). In specific, microglia can self-activate and secrete various substances that exacerbate or alleviate the neuroimmune response to TBI. In addition, microRNAs (miRNAs) are involved in the functional regulation of microglia. However, molecular markers that reflect the dynamics of TBI have not yet been found in peripheral tissues.

**Methods:**

Paired samples of peripheral blood were collected from patients with TBI before and after treatment. Next-generation sequencing and bioinformatics analysis were used to identify the main pathways and biological functions of TBI-related miRNAs in the samples. Moreover, lipopolysaccharide-treated human microglia were used to construct a cellular immune-activation model. This was combined with analysis of peripheral blood samples to screen for highly expressed miRNAs derived from activated microglia after TBI treatment. Quantitative reverse-transcriptase polymerase chain reaction was used to determine the expression levels of these miRNAs, allowing their relationship with the severity of TBI to be examined. Receiver operating characteristic (ROC) curves were constructed to analyse the clinical utility of these miRNAs for determining the extent of TBI.

**Results:**

Sequencing results showed that 37 miRNAs were differentially expressed in peripheral blood samples from patients with TBI before and after treatment, with 17 miRNAs being upregulated and 20 miRNAs being downregulated after treatment. The expression profiles of these miRNAs were verified in microglial inflammation models and in the abovementioned peripheral blood samples. The results showed that hsa-miR-122-5p and hsa-miR-193b-3p were highly expressed in the peripheral blood of patients with TBI after treatment and that the expression levels of these miRNAs were correlated with the patients’ scores on the Glasgow Coma Scale. ROC curve analysis revealed that abnormally high levels of expression of hsa-miR-122-5p and hsa-miR-193b-3p in peripheral blood have some clinical utility for distinguishing different extents of TBI and thus could serve as biomarkers of TBI.

**Conclusion:**

Abnormally high levels of expression of hsa-miR-122-5p and hsa-miR-193b-3p in the peripheral blood of patients with TBI were due to the activation of microglia and correlated with the severity of TBI. This discovery may help to increase understanding of the molecular pathology of TBI and guide the development of new strategies for TBI therapy based on microglial function.

**Supplementary Information:**

The online version contains supplementary material available at 10.1186/s40001-024-01790-y.

## Introduction

Traumatic brain injury (TBI) occurs when the head is subjected to impact or external force that damages normal brain function or structure [1]. Depending on individual factors, similar impacts may cause different injuries in different populations. Therefore, TBI exhibits significant heterogeneity, with short-term and long-term outcomes influenced by specific levels of intracranial injury, presence of extracranial injury, age, and existing comorbidities [2]. TBI is the main cause of death and disability in individuals under the age of 45, and neuroimmune responses play a crucial role in the pathological process of brain injury repair [1, 2]. Microglia are the resident immune cells of the brain and rapidly change their state based on their microenvironment. Various degrees of TBI can rapidly activate microglia, and their activation and proliferation are important manifestations of inflammatory responses of the central nervous system [3].

MicroRNAs (miRNAs) are members of the non-coding RNA family [4] that are usually 17–24 nucleotides (nt) long and play a role in post-transcriptional gene regulation by pairing with complementary sequences of target genes [5]. miRNAs also play an important role in the pathological progression of various nerve injuries [4, 6, 7]. Due to the low molecular weight of miRNAs, they are easily distributed and detected in various bodily fluids. Humoral miRNAs secreted by microglia are crucial for promoting neurite outgrowth and synaptic repair [8, 9]. Moreover, several miRNAs hold promise as biomarkers and potential therapeutic tools for neurological diseases [6, 10]. Recent studies found that the expression levels of some miRNAs in patients with TBI and in animal models of TBI differ from those in healthy controls. Ge et al. determined that the expression level of miR-124-3p was significantly increased in the exosomes of microglia in animal models of TBI. Moreover, they observed that increasing the expression level of miR-124-3p in the hippocampus helped to reduce neurodegeneration and improve impaired cognitive function [8]. Wang et al. performed miRNA sequencing of extracellular vesicles and found that miR-20b-5p was highly expressed in mice with TBI and under mild hyperthermia. They also determined that miR-20b-5p directly targets phosphatase and tensin homologue and activates the phosphoinositide-3-kinase–AKT pathway, thereby playing a preclinical therapeutic role in promoting neurorehabilitation [11]. One of the most common computed tomography (CT) manifestations in patients with TBI is subarachnoid haemorrhage (SAH), which is seen in approximately 30–40% of patients with moderate-to-severe TBI and 5% of patients with mild TBI [1, 2]. Compared with patients with moderate or severe TBI without SAH, such patients with SAH have poorer outcomes. In addition, the circulating miRNA expression profiles of patients with SAH were found to be significantly different from those of healthy controls [12]. Furthermore, intravenously injected exosomes containing miR-193b-3p showed neuroprotective and anti-inflammatory effects in a mouse model of SAH induced by brain injury [9].

In the current study, we used small-RNA high-throughput sequencing technology to screen differentially expressed miRNAs in peripheral blood samples collected from patients with TBI before and after they had received treatment. Subsequently, we conducted bioinformatics analyses to summarise the biological pathways and mechanisms of these differentially expressed miRNAs. The results increase understanding of the molecular pathology and peripheral biomarker changes that occur during the recovery period of TBI.

Lipopolysaccharide (LPS) is a structural component of the outer membranes of Gram-negative bacteria that can induce neuroinflammatory states by promoting the activation of microglia, leading to the release of pro-inflammatory cytokines and a series of neurotoxic factors [13, 14]. We determined that multiple TBI-associated circulating miRNAs were highly expressed in LPS-treated microglia. We hypothesised that some of these abnormally expressed miRNAs reflect the extent of TBI. If so, this would increase our understanding of the molecular pathological roles of microglia-associated miRNAs related to TBI. Further analysis of the clinical utility of these miRNAs in diagnosing and assessing TBI may support the development of new clinical treatments for TBI.

## Methods

### Clinical specimens

Fifty pairs of peripheral blood samples were collected from patients with TBI. in specific, a 1 mL sample of peripheral blood was drawn from patients within 30 min after they had been diagnosed with a TBI in our department, and another 1 mL sample was drawn from these patients after they had received 2 weeks of treatment. The patients ranged in age from 18 to 60 years.

All blood collection vessels were pre-treated with sodium citrate anticoagulant. The collected blood samples were immediately stored in an ultralow-temperature refrigerator at −80 ℃. All patients met the diagnostic criteria for TBI specified in the 11th revision of the International Classification of Diseases. The patients had no history of neurological or psychiatric disorders, substance abuse, severe liver and kidney dysfunction, or recent use of glucocorticoids. In addition, they were all male and none had any infectious diseases.

### Small RNA sequencing

Five pairs of matched (i.e., pre-treatment and post-treatment) peripheral blood samples were subjected to small-RNA sequencing. Total RNA was extracted from samples using the TRIzol method and then separated by polyacrylamide gel electrophoresis (PAGE). Subsequently, small RNA was recovered by selecting bands comprising 18–30 nt. Next, each end of a small RNA was joined to a 3′ or a 5′ connector, as appropriate, and the resulting modified small RNA was reverse-transcribed and then amplified by PCR. The resulting DNA was separated by PAGE and then the band comprising approximately 140 bp was dissolved in a solution of ethidium bromide. The resulting mixture was used to complete library construction. The constructed library was tested for quality and yield using an Agilent2100 and ABI StepOnePlus Real Time PCR System (Life Technologies, Santa Clara, CA, United States), and then sequenced on an Illumina NovaSeq 6000 platform. We subjected the original disembarkation data to the following filtering process to obtain high-quality small RNA data: (a) remove the 5′ connector, filter out reads without the 3′ connector, and retain the sequence before the 3′ connector; (b) filter out low-quality reads (those with a mass < 20 or more than one base number) to obtain high-quality reads; (c) filter out reads without an insert fragment and those with an insert fragment of less than 18 nt; (d) filter out reads containing polyadenine (i.e., reads in which more than 70% of the bases were adenine). The resulting clean small RNA sequences were used in subsequent analyses.

### Bioinformatics analysis

Basic Local Alignment Search Tool 2.2.25 was used to screen for small RNA sequences with an identity greater than 97%. Those that met this condition were aligned with ribosomal RNAs (rRNAs), small conditional RNAs (scRNAs), small nucleolar RNAs (snoRNAs), small nuclear RNAs (snRNAs), and transfer RNAs (tRNAs) selected from GenBank and Rfam databases. This allowed as many rRNAs, scRNAs, snoRNAs, snRNAs, and tRNAs as possible to be identified and then removed from the samples. Subsequently, due to the unique secondary structure of miRNAs, we were able to align small RNA sequences with a reference genome and then use secondary structure prediction to infer the potential existence of new miRNAs.

As this was an miRNA omics study, principal component analysis (PCA) was used to reduce information consisting of thousands of dimensions (i.e., the expression levels of thousands of miRNAs in the samples) to comprehensive indicators consisting of several dimensions (i.e., principal components). This facilitated inter-sample comparisons and ensured that the information contained in the original data was retained as much as possible. We used R (http://www.r-project.org/) for PCA, and constructed a two-dimensional coordinate plot based on the values of each sample in the first principal component (PC1) and the second principal component (PC2).

We conducted a heatmap analysis of all miRNAs, namely existing miRNAs, known miRNAs, new miRNAs, and the first type of miRNA. In specific, with reference to miRNA expression levels, we performed hierarchical clustering analyses of the relationship between samples and miRNAs, and depicted the clustering results in heatmaps. The hierarchical clustering analyses examined the expression levels of miRNAs in different samples and their corresponding genes. Each column in the heatmap represented a sample, and each row represented an miRNA. The expression levels of miRNAs in different samples were represented by different colours, with red indicating higher expression levels than normal and green indicating lower expression levels than normal. Subsequently, we conducted Gene Ontology (GO) and Kyoto Encyclopedia of Genes and Genomes (KEGG) enrichment analyses of the differentially expressed miRNA target genes in each sample. First, we input the miRNA target genes to the GO term mapping database (http://www.geneontology.org/). Then, we calculated the number of miRNA target genes matching each term and thereby obtained a GO function list of miRNA target genes. Next, we applied hypergeometric tests to identify GO entries that were significantly enriched in miRNA target genes compared with the background. Subsequently, we conducted significant enrichment analysis using KEGG PATHWAY to identify the most important biochemical metabolic pathways and signal transduction pathways in which the miRNA target genes were involved.

### Cell culture

The cell line HMO6 is derived from human brain microglia and is commonly used to experimentally study the function and characteristics of these cells. HMO6 cells have multi-directional differentiation ability and can differentiate into various types of neural cells, such as neurons, astrocytes, and oligodendrocytes. In the current study, HMO6 cells were cultured in a T25 culture bottle in a medium consisting of a mixture of 0.5% penicillin–streptomycin, 90% high-glucose Dulbecco’s modified Eagle medium, and 10% foetal bovine serum, and the medium was changed every 2–3 days. HMO6 cells with logarithmic growth were seeded onto a six-well plate, and the wells were randomly divided into a control group and an intervention group. The latter group was treated with LPS (150 ng/mL) to establish a cellular model of the inflammatory response, as LPS is an immune stimulator that induces cellular inflammation and immune responses. All cells were maintained in a cell culture incubator in an atmosphere of moist air with 95% relative humidity containing 5% carbon dioxide at 37 ℃.

### RNA extraction and qRT-PCR analysis

TRIzol Reagent (Ambion, Carlsbad, CA, USA) was used to isolate total RNA from HMO6 cells. A Multiskan GO1510 spectrophotometer (Thermo Fisher Scientific, Vantaa, Finland) was used to measure the quantity and purity of RNA, and a CFX Connect Real-Time PCR Detection System (Bio-Rad, USA) was used to perform real-time quantitative reverse-transcription polymerase chain reaction (qRT-PCR) analyses. First, the miRNA expression in HMO6 cells was evaluated using a miDETECT A Track™ miRNA qRT-PCR Starter Kit (RiboBio, Guangzhou, China), as described previously [15, 16]. Primers for hsa-miR-122-5p, hsa-miR-193b-3p, and U6 were designed and synthesised by RiboBio. All researchers were blinded to the clinical and pathological diagnoses of the patients from whom the samples had been collected. The target miRNAs and an endogenous control gene (U6) were amplified in triplicate wells. Finally, relative expression levels of miRNAs were calculated using the 2^−△△Ct^ method [17].

### Clinical severity assessment

The Glasgow Coma Scale (GCS) is widely used to objectively assess a patient’s level of consciousness on a scale from 3 (completely unresponsive) to 15 (responsive). GCS scores have also been widely used to grade TBI severity and prognosis, and were found to be negatively correlated with the positive rate of detection of TBI by CT. For example, the rate of intracranial injury and the need for neurosurgical treatment of patients with a GCS score of 14 are double that of patients with a GCS score of 15. The relationships between GCS scores and TBI severity are used to classify TBI into three grades: mild TBI (GCS score = 13–15; mortality = 0.1%), moderate TBI (GCS score = 9–12, mortality = 10%) and severe TBI (GCS score < 9, mortality = 40%). This study examined the correlation between the patients’ GCS scores and the levels of expression of TBI-associated miRNAs in their peripheral blood samples.

### Receiver operating characteristic (ROC) curve analysis

An ROC curve, also known as a sensitivity curve, is an analytical tool created by drawing coordinate schemata on a two-dimensional plane [18, 19]. Recently, ROC curve-based approaches were tailored to and applied in machine learning and data-mining. In an ROC curve, the horizontal coordinate is the false positive rate (FPR) and the vertical coordinate is the true positive rate (TPR) [20]. Moreover, the area under an ROC curve, commonly known as the area under the curve (AUC), can be used to indicate the performance of the classifier of interest. For example, an ROC curve in which 0.5 < AUC < 1 indicates that the corresponding classifier performs better than a random guess, and the higher the AUC, the higher the accuracy of the classifier. In the current study, ROC curves were used to evaluate whether miRNA expression levels in peripheral blood had clinical utility for the diagnosis of TBI.

### Statistical analysis

Statistical analyses were performed using Statistical Products and Service Solutions (SPSS) 17.0 software (SPSS, Chicago, IL, USA). Paired *t* tests were used to analyse the differences between the expression levels of miRNAs in patients with TBI before they had received treatment and those in such patients after they had received treatment. Student’s *t* tests were used to analyse the differences between the experimental and control groups. Single-factor analyses of variance were used to compare data between three or more groups. A *P* value of less than 0.05 was considered to indicate a statistically significant difference.

## Results

### Identification of miRNAs in peripheral blood sample of patients with TBI before and after treatment

**Fig. 1:**
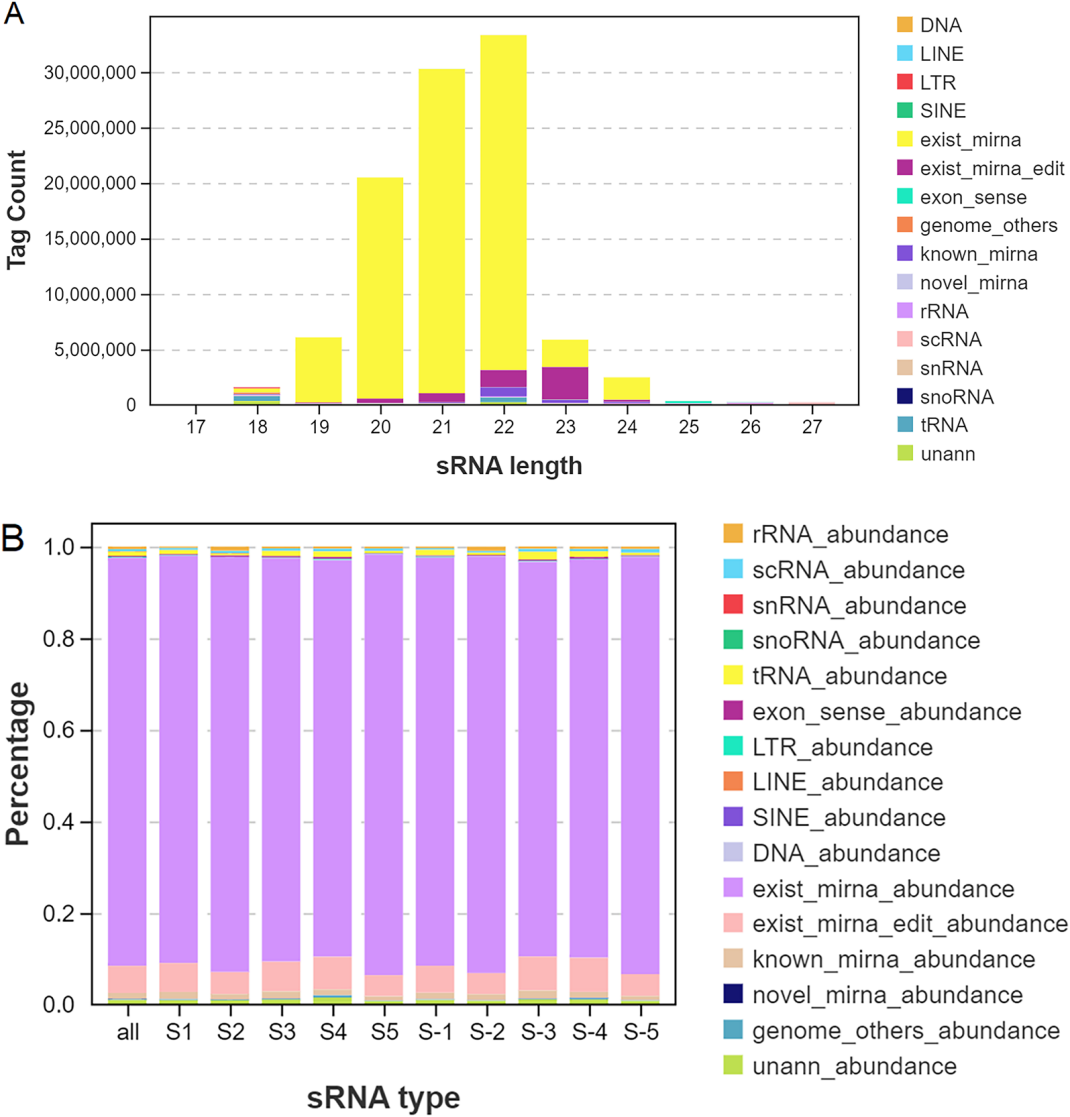
Identification of miRNAs in peripheral blood of patients with TBI before and after they had received treatment, respectively. **A** Length distribution of the various types of sRNA in each sample. **B** Statistics on the proportions of sRNA types in each sample

The length distribution of tag sequences obtained from small RNA sequencing follows certain rules, and thus by determining the length distributions of tag sequences in data, one can obtain an approximate understanding of the quality of the data. In the current study, the tag sequences in the peripheral blood samples of patients with TBI were a maximum of only 22 bp in length (Fig. 1A), which is consistent with the distribution patterns of tag lengths in conventional animal samples. Statistical analysis of the sources of the identified small RNAs revealed that the vast majority were derived from known miRNAs and that there was no significant between-sample difference in their content of small RNAs (Fig. 1B). There was also no significant difference between the total number of miRNAs in the peripheral blood samples of patients with TBI before and after they had received treatment, respectively (Additional file 1: Fig. S1).

### Sample association analysis and screening of samples for miRNAs related to TBI

**Fig. 2:**
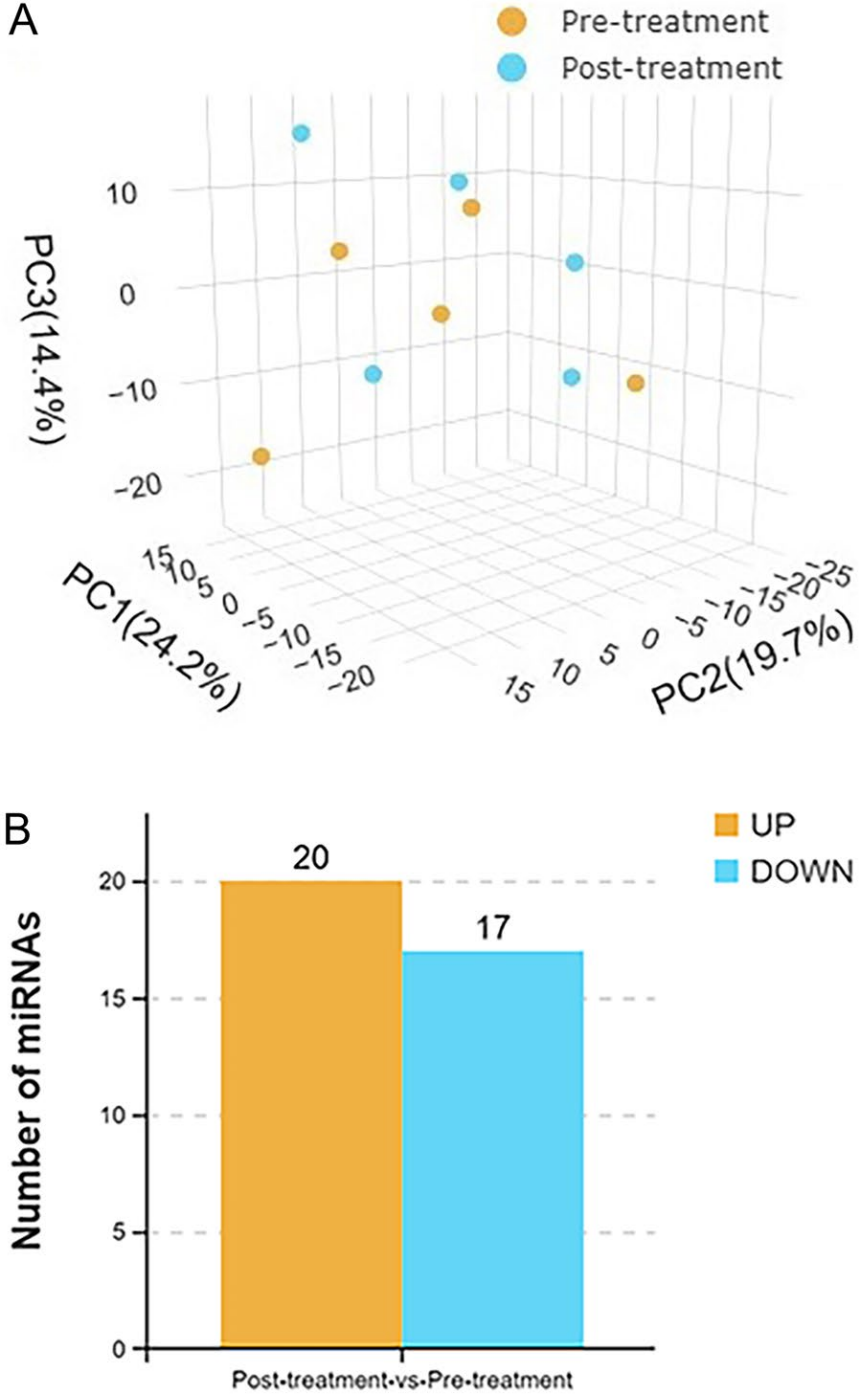
Sample relationship analysis and differential miRNA screening of peripheral blood samples of patients with TBI before and after they had received treatment, respectively. **A** PCA generated using R based on miRNA expression information. The values in the axis label brackets represent the percentage of the total variance explained by the principal component. **B** Numbers of differentially expressed miRNAs in peripheral blood that met the screening threshold in each group before and after they had received treatment, respectively. The sequencing results showed that after they had received treatment, the expression levels of 20 miRNAs had increased and those of 17 miRNAs had decreased in the peripheral blood samples of patients with TBI

PCA can reduce information in tens of thousands of dimensions (i.e., the expression levels of tens of thousands of genes) contained in a sample into comprehensive indicators (principal components) in several dimensions, thereby facilitating comparison of samples, and analyses of repeatability and differences between samples. There were no significant changes in the principal components of the patients’ peripheral blood samples after they had received treatment for TBI, which confirmed the reliability of the obtained data (Fig. 2A). After paired analysis of sequencing data from five samples, it was found that 20 miRNAs were upregulated and 17 miRNAs were downregulated in the peripheral blood of patients with TBI after they had received treatment (Fig. 2B).

### Identification of differentially expressed miRNA profiles of peripheral blood samples of patients with TBI

**Fig. 3:**
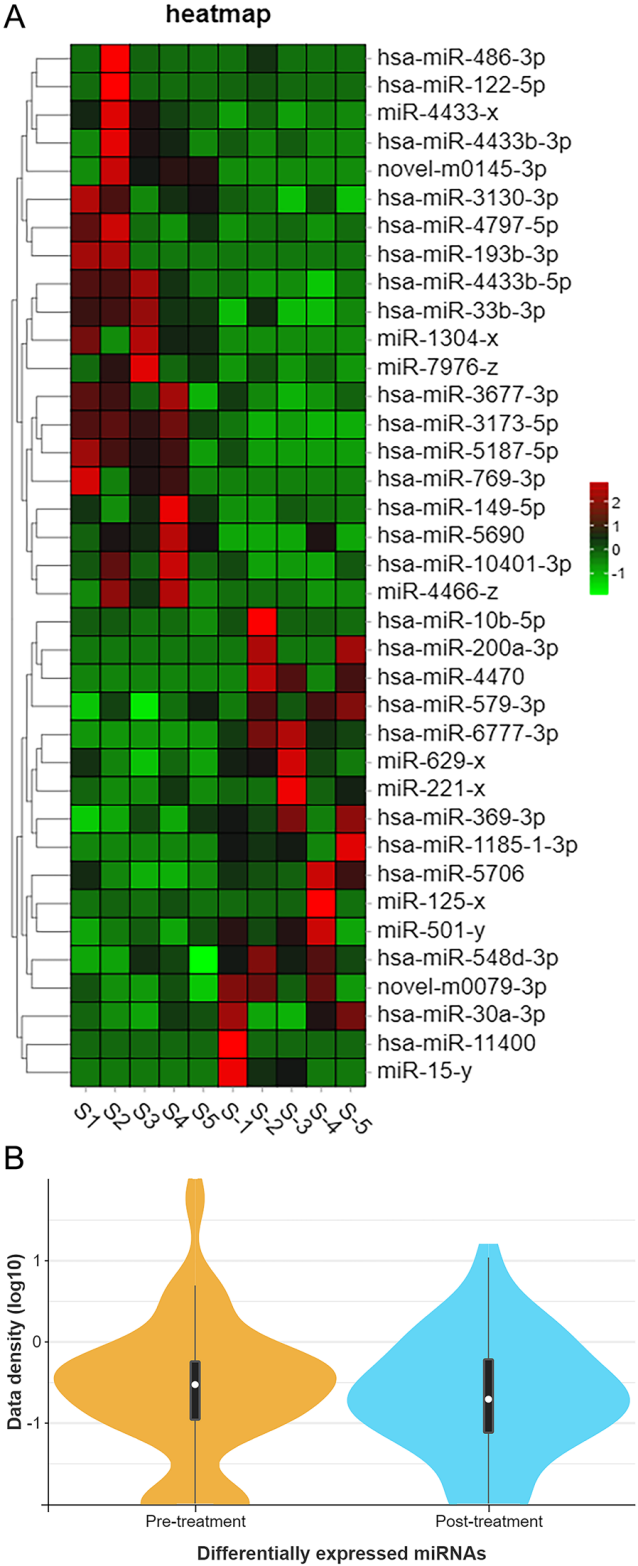

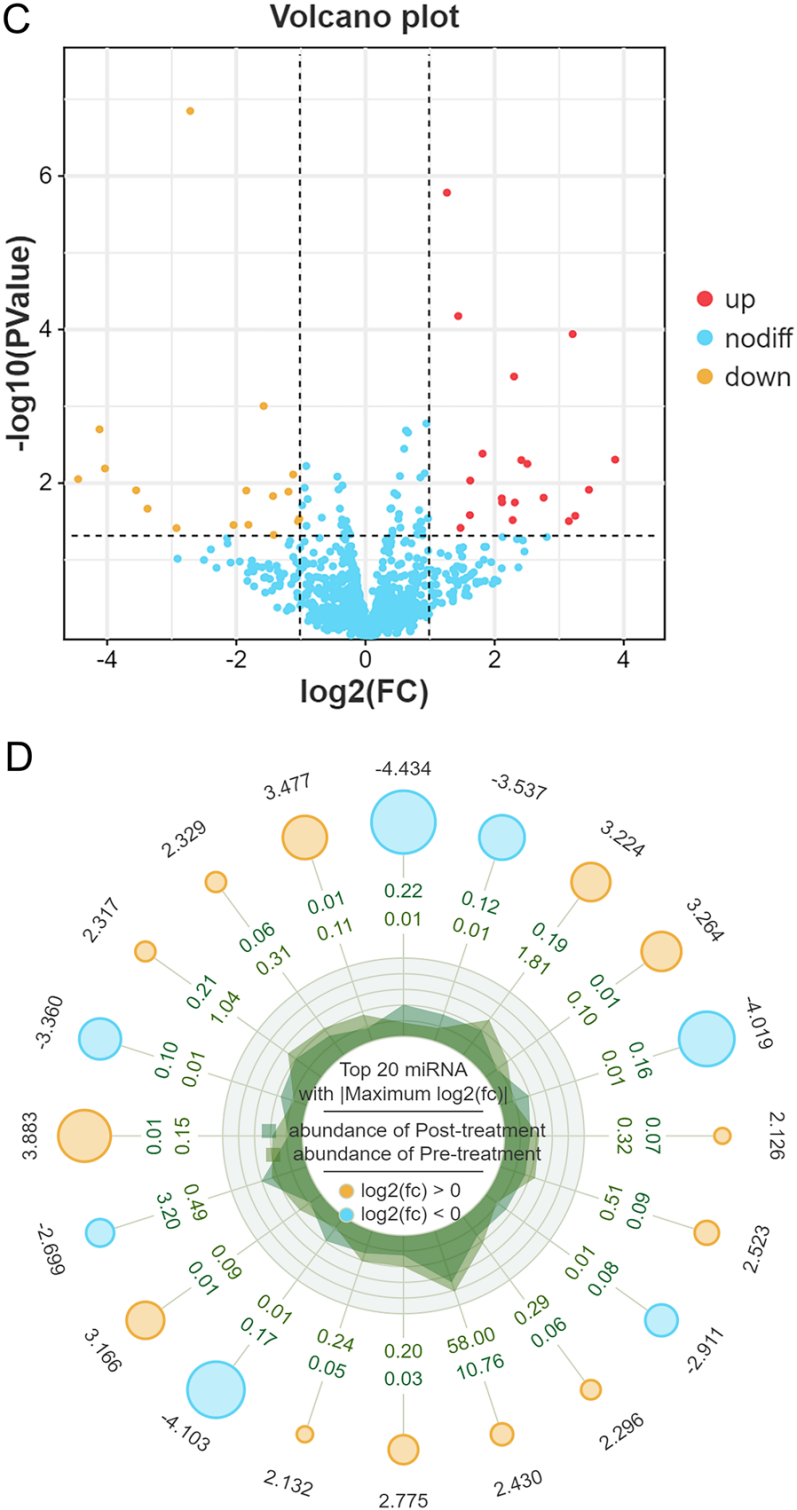
Biometric analysis of differential expression of miRNAs in peripheral blood of patients with TBI before and after they had received treatment, respectively. **A** Heat map of the miRNAs expressed in the peripheral blood of patients with TBI. S1 to S5: pre-treatment; S-1 to S-5: post-treatment (**B**) Violin plots showing density of differentially expressed miRNAs in each group. **C** Volcano maps showing miRNAs with a differential expression. **D** Radar chart displaying the information of the gene with the greatest overall differential expression (based on *P* values)

The expression profiles of miRNAs in samples of the peripheral blood of patients with TBI before and after they had received treatment are shown in Additional file 6: Table S1. Significant differences in the expression levels of miRNAs in each sample were depicted in a heat map to reveal between-sample variations in miRNA expression patterns and allow cluster analysis of samples and miRNAs based on miRNA expression levels (Fig. 3A). The data densities of differential miRNAs within each group were depicted in a violin plot (Fig. 3B). The differential expression of miRNAs between the two groups was depicted in a volcano plot. The horizontal axis represents the logarithmic value of the multiple of differential expression in expression, and the vertical axis represents the negative logarithm of multiple of differential expression in the false discovery rate (FDR). Different colours represent the upregulated and downregulated miRNAs, as determined from screening based on the thresholds of expression, and the blue dots represent no differences. Thus, in the plot, the closer a point is to the baseline on both axes, the greater the between-group difference in the expression of the miRNA it represents (Fig. 3C). Information on the miRNAs with the greatest overall between-group differences in expression, based on *P* or *Q* values, was displayed in a radar chart (Fig. 3D).

### GO enrichment of differentially expressed miRNA target genes

**Fig. 4:**
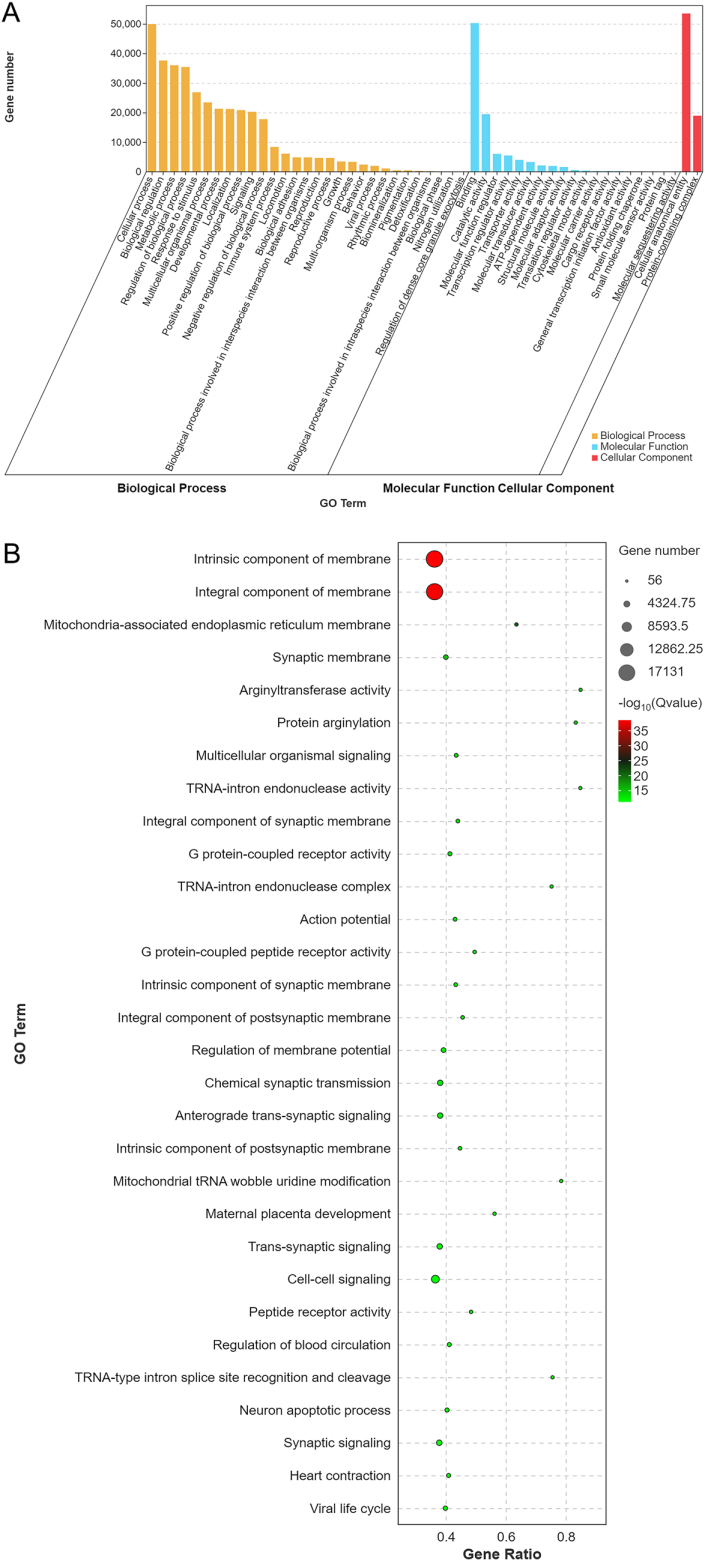

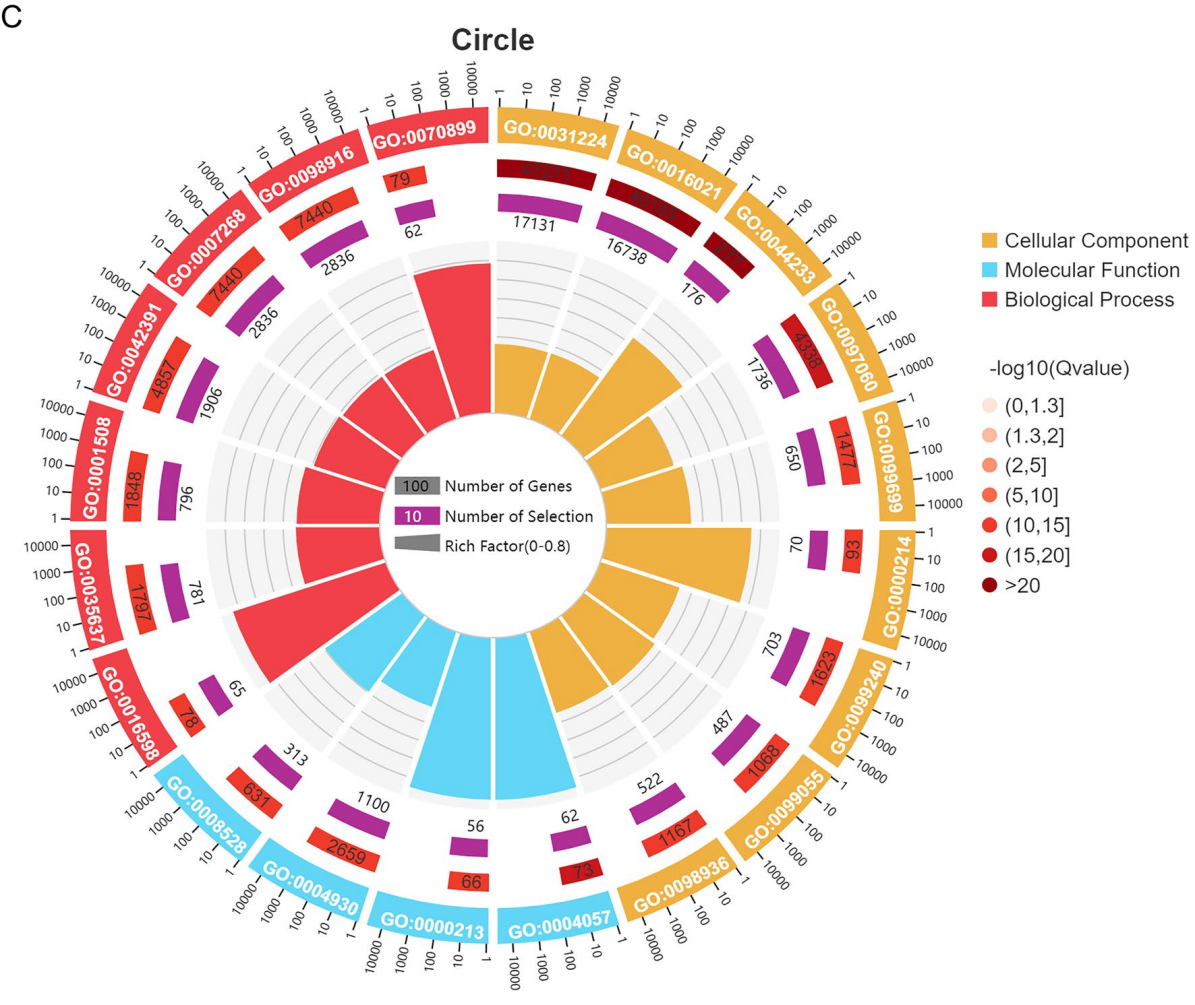
GO enrichment analysis of differentially expressed miRNA target genes. **A** Secondary bar chart classification of differentially expressed genes based on the GO database. **B** Bubble plots showing significantly enriched target genes in the GO database. **C** Enrichment circle diagram of differentially expressed miRNA target genes

A second-level classification bar chart was constructed based on the GO database and used for enrichment analysis of target genes to detect upregulated and downregulated differential miRNAs and for statistical analysis of the numbers of enriched pathways and included genes (Fig. 4A). Significant enrichment bar charts and GO enrichment bar charts were constructed and used to select the GO terms that were most enriched, based on *P* values or FDR values, or to draw graphs based on custom GO terms (Additional file 2: Fig. S2a, Additional file 3: Fig. S2b). A GO enrichment bubble plot was constructed using the 20 GO terms with the lowest *Q* values. GO terms are listed on the vertical axis, and enrichment factors are listed on the horizontal axis, where an enrichment factor is the number of miRNA target genes in a GO term divided by the numbers of miRNA target genes in all of the GO terms. The size of a bubble is proportional to the number of target genes, and the redder the colour, the smaller the *Q* value (Fig. 4B). The first circle represents the top 20 most enriched GO terms, and the outer circle represents the coordination number of the miRNA target genes. Different colours represent different ontologies. The second circle represents the number and *Q* value of the GO term in the miRNA target gene background. The third circle represents the number of miRNA target genes in the GO term. The fourth circle represents the enrichment (Rich) factor value of each GO term, which is the number of miRNA target genes in that GO term divided by the number of all genes in that GO term. Each cell in the background grid represents 0.1 (Fig. 4C).

### KEGG enrichment of differentially expressed miRNA target genes

**Fig. 5:**
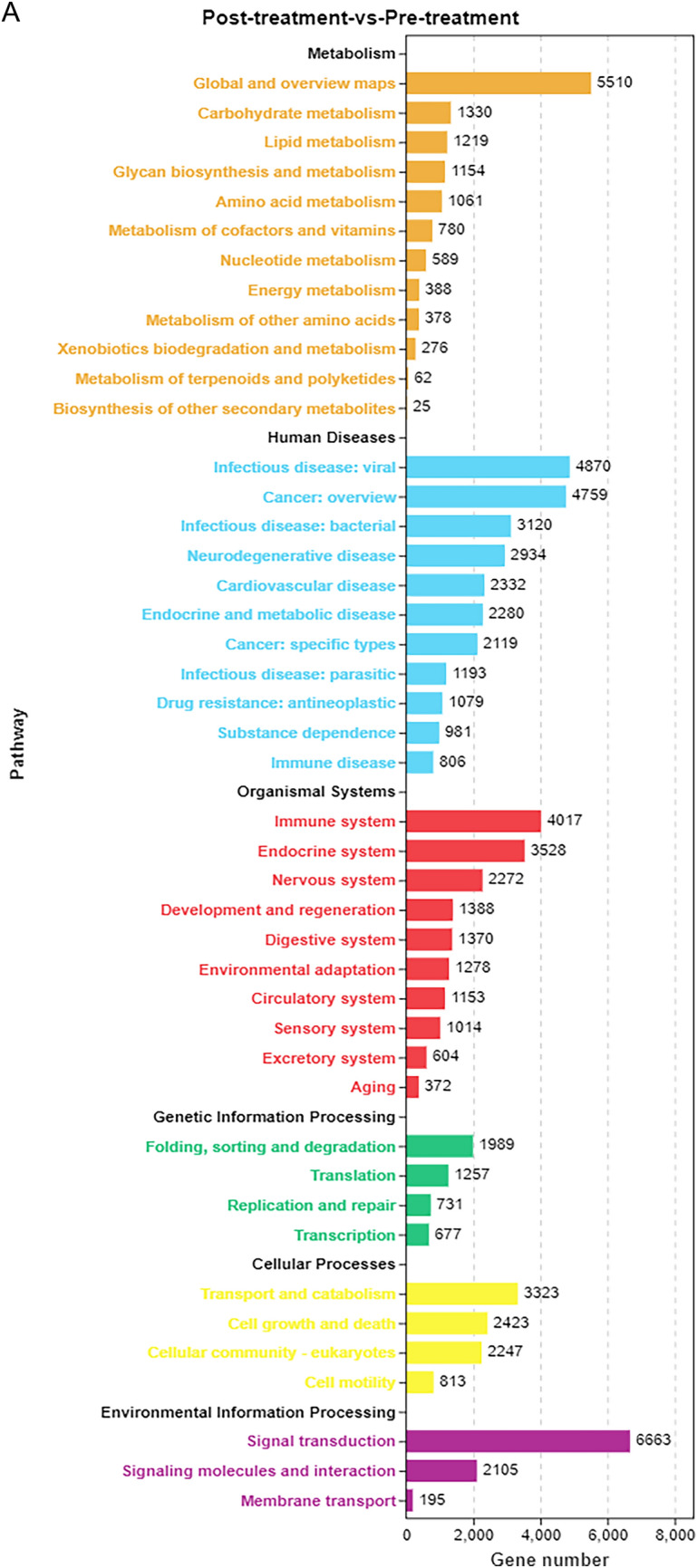

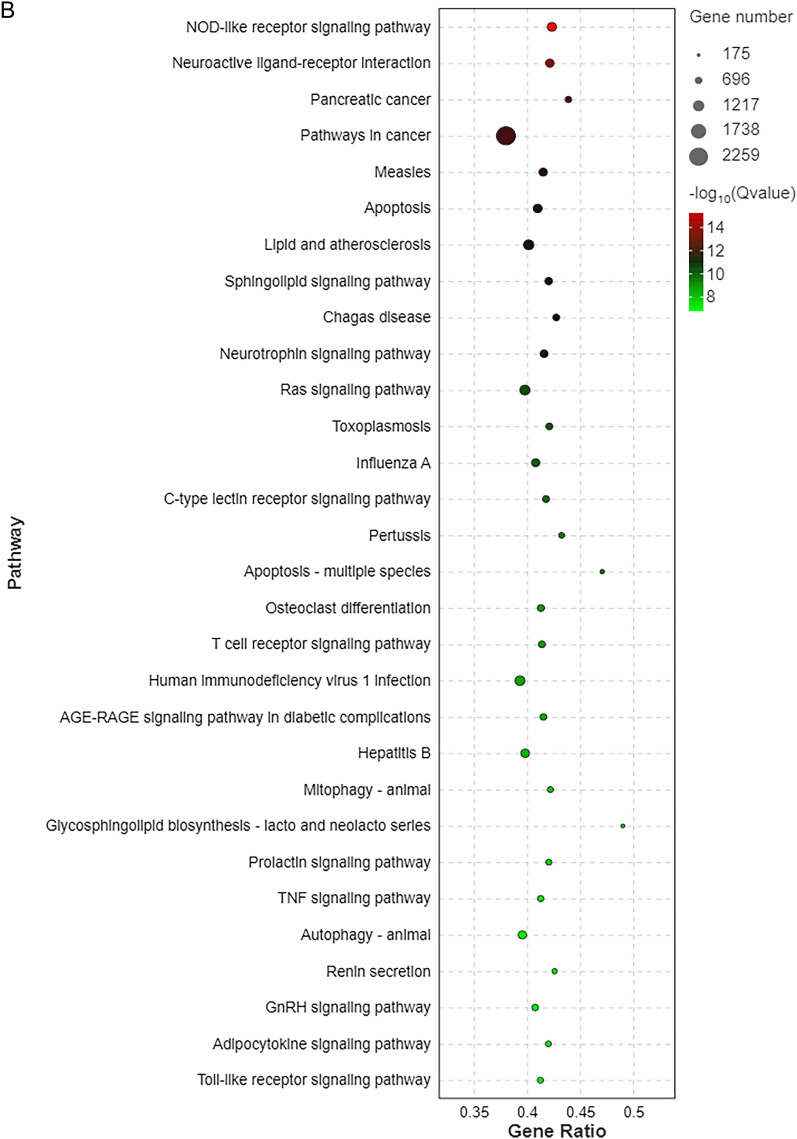

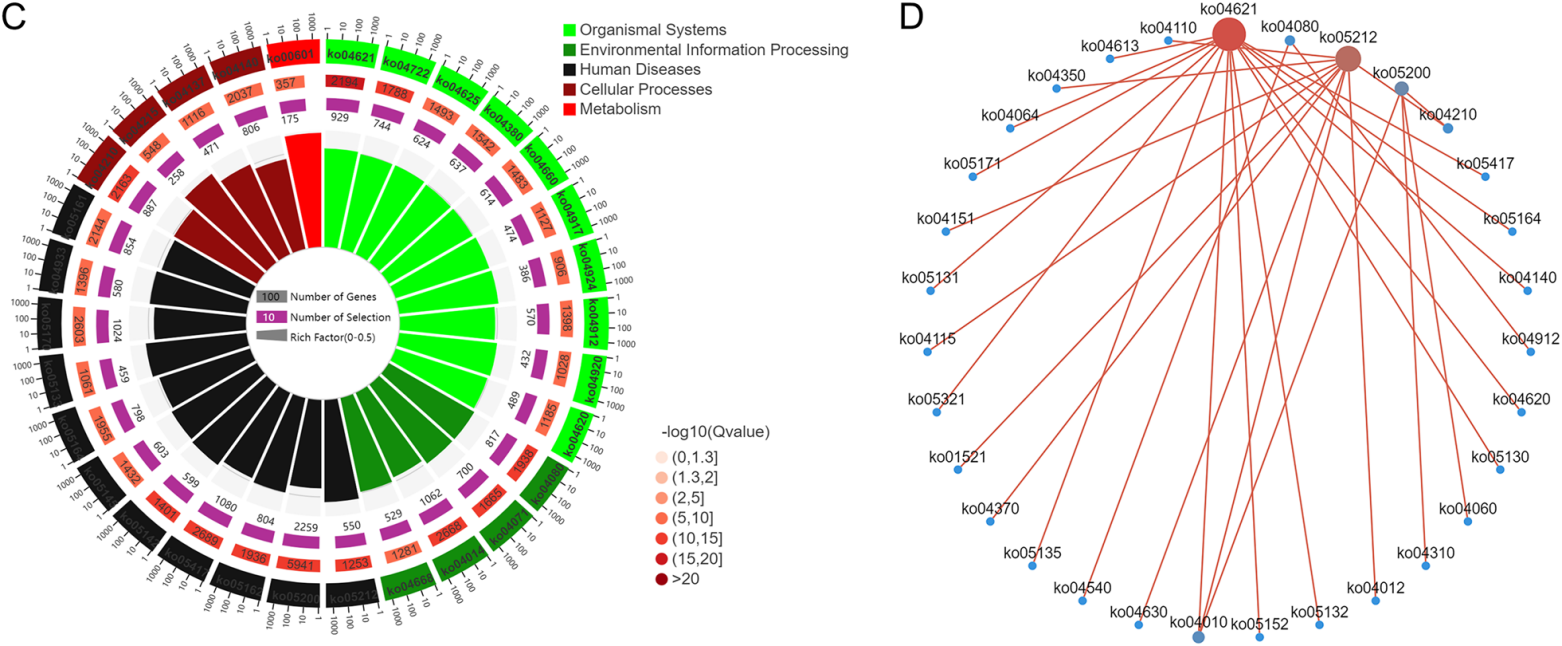
KEGG enrichment analysis of differentially expressed miRNA target genes in in the peripheral blood of patients with TBI. **A** Enrichment pathways and statistics of target genes in KEGG taxonomic units. **B** Bubble plot of the significance of target gene enrichment pathways in the KEGG. **C** Enrichment circle diagram of target genes in the KEGG. **D** Network diagram of enriched pathways of target genes in the KEGG

A statistical chart depicting an enrichment analysis of differentially expressed miRNA target genes was constructed, with the number of enriched pathways and included genes calculated based on the first- and second-level classifications of the KEGG database (Fig. 5A). In addition, a significance bar chart was constructed to graphically depict the most enriched pathways based on *P* values or FDR values, or to depict a custom pathway (Additional file 4: Fig. S3A). Furthermore, a KEGG enrichment bar chart was constructed using the 20 pathways with the lowest *Q* values (Additional file 5: Fig. S3B). In addition, a KEGG enrichment bubble map was constructed using the 20 pathways with the lowest *Q* values. The pathways are represented on the ordinate, and the Rich factor is represented on the horizontal coordinate and indicates the number of miRNA target genes in the pathway divided by the total number of genes in this pathway. The size of the bubble represents the number of target genes, and the redder the colour, the smaller the *Q* value (Fig. 5B). The first circle in the KEGG enrichment diagram represents the pathway of the top 20 most enriched genes, and the coordinates outside the circle are the number of miRNA target genes. The second circle represents the number of miRNA target genes belonging to this pathway and their *Q* values. In this classification, the larger the number of miRNA target genes, the longer the bars; and the smaller the *Q* value, the redder the colour. The third circle represents the number of miRNA target genes in the pathway. The fourth circle represents the Rich factor of each pathway, that is, the number of miRNA target genes in the pathway divided by the total number of genes in the pathway. Each cell in the background grid represents 0.1 (Fig. 5C). Moreover, a KEGG enrichment network diagram of differentially expressed miRNA target genes was drawn based on the information of pathways enriched in differentially expressed genes and by utilising the interactive relationships between pathways to assist in searching for core pathways (Fig. 5D).

### LPS-promoted upregulation of TBI-associated miRNA expression in HMO6 cells

**Fig. 6:**
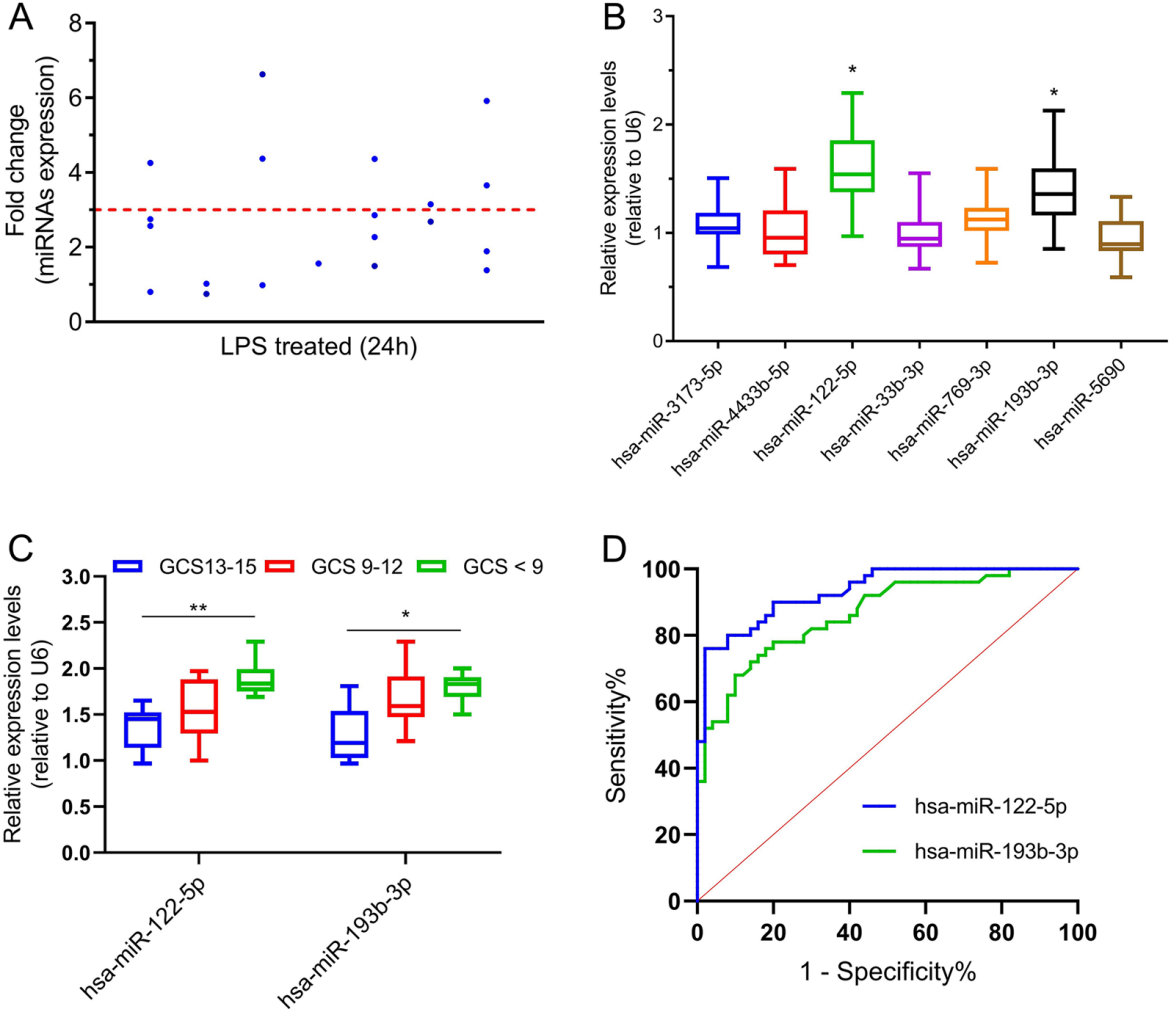
Clinical value of differentially expressed miRNAs in peripheral blood of patients with TBI. **A** Seven miRNAs were upregulated in LPS-treated human microglia. **B** The peripheral blood of TBI patients showed significantly higher levels of expression of hsa-miR-122-5p and hsa-miR-193b-3p after the patients had received treatment than before they had received treatment. **C** The levels of differentially expression miRNAs in the peripheral blood of patients with TBI were correlated with TBI severity. **D** The levels of expression of hsa-miR-122-5p and hsa-miR-193b-3p in peripheral blood were of some utility for supporting a diagnosis of TBI. *P < 0.05, **P < 0.01, *** P < 0.001

TBI-associated miRNAs were significantly overexpressed in LPS-treated HMO6 cells. In particular, the expression of seven miRNAs (hsa-miR-3173-5p, hsa-miR-4433b-5p, hsa-miR-122-5p, hsa-miR-769-3p, hsa-miR-33b-3p, hsa-miR-193b-3p, and hsa-miR-5690) was increased more than threefold in LPS-treated HMO6 cells compared with controls (Fig. 6A). This suggests that the high levels of expression of these miRNAs are associated with the inflammatory activation state of microglia.

### Expression levels of hsa-miR-122-5p and hsa-miR-193b-3p were significantly upregulated in the peripheral blood of patients with TBI after they had received treatment

To validate the results of the above-described small RNA sequencing analysis and LPS screening, we analysed 50 pairs of peripheral blood samples from patients with TBI that were collected before and after they had received treatment. The expression levels of the above-mentioned seven miRNAs were determined in these samples, and hsa-miR-122-5p and hsa-miR-193b-3p were found to be significantly highly expressed (Fig. 6B).

### Evaluation of clinical utility of miRNAs in assessment of TBI

The GCS score is an important indicator of the severity of TBI. Thus, we examined whether there was a correlation between the levels of expression hsa-miR-122-5p and hsa-miR-193b-3p in the peripheral blood of patients with TBI and their GCS scores. The results showed a negative correlation between the aforementioned factors, with increased levels of expression of hsa-miR-122-5p and hsa-miR-193b-3p reflecting moderate or high levels of TBI (Fig. 6C). Furthermore, ROC curve analysis revealed that hsa-miR-122-5p and hsa-miR-193b-3p can, to a certain extent, serve as indicators of the presence of TBI (hsa-miR-122-5p, AUC = 0.9334, *P* < 0.001; hsa-miR-193b-3p, AUC = 0.8642, *P* < 0.001) (Fig. 6D and Additional file 7: Table S2).

## Discussion

Various TBIs can rapidly activate microglia, and their activation and proliferation are important manifestations of central nervous system inflammatory responses [21, 22]. However, these inflammatory reactions are a ‘double-edged sword’. In response to stimuli such as injury and trauma, rapidly activated microglia undergo varying degrees of pathological change, and their immune function also undergoes a series of changes [22]. They can release many immune regulatory factors within a few minutes after brain injury that mediate a series of cascading inflammatory reactions, such as those associated with the expression of adhesion molecules, cell infiltration, and secretion of inflammatory molecules, which collectively lead to the aggravation of brain injury [22–24]. Nonetheless, the activation of microglia is a protective response to harmful factors. That is, neurotrophic factors produced by microglia promote direct and mutual information exchange between the nervous and immune systems, and have neuroprotective effects [22–24]. For example, these factors help to clear extracellular oxidative proteins, engulf cell debris, and degenerate myelin sheaths in the brain, thereby providing a suitable microenvironment for neurons and promoting neural tissue repair [22].

In the current study, we used next-generation sequencing technology to screen for miRNAs that were highly expressed in the peripheral blood of patients with TBI after they had received treatment (Figs. 1, 2, 3). Bioinformatics analysis showed that most of the target genes of miRNAs that remained abnormally expressed after treatment in these patients are involved in cell growth and neural function recovery (Figs. 4, 5). In addition, analyses of clinical TBI samples and microglial inflammation models revealed that the expression of these target genes is highly likely to be triggered by the activation of microglia after TBI and to some extent reflect the severity of TBI (Fig. 6).

Our extraction of miRNAs from samples of patients’ peripheral whole blood had certain advantages. For example, the miRNAs in peripheral blood are composed of two parts: nucleated miRNAs and free circulating miRNAs. Due to the presence of the blood–brain barrier, it is difficult to detect changes in immune factors within the vast majority of the brain and thus it is difficult to identify immune factors that could be used as biomarkers of disease [9, 25]. However, miRNAs are encapsulated in exosomes secreted by central nervous system cells and these exosomes cross the blood–brain barrier to perform long-range cellular communication functions [9, 26]. Subsequently, these miRNAs enter the circulatory system and be easily detected [9, 27]. Lai et al. and Sheng et al. independently found that the expression of hsa-miR-193b-3p in the exosomes of patients with TBI was significantly higher than that in the exosomes of healthy controls [9, 12]. In addition, the injection of exosomes containing hsa-miR-193b-3p was found to significantly attenuate neuroinflammation in animal models of TBI [9]. In the current study, we found that the level of expression of hsa-miR-193b-3p in peripheral blood remained high in patients with TBI who had received treatment. This suggests that circulating levels of hsa-miR-193b-3p can to some extent reflect the neuroimmune status of the brain of patients with TBI. In addition, some nucleated cells in the peripheral blood produce an immune response to bodily damage by secreting exosomes containing abnormal levels of miRNAs, thereby enabling communication between different cells and regulating the inflammatory state. Li et al. reported that exosomes containing miR-122-5p secreted by LPS-induced neutrophils regulated the apoptosis and permeability of brain microvascular epithelial cells [13]. In the current study, high-throughput sequencing revealed that there were high levels of hsa-miR-122-5p expressed in the peripheral blood of patients with TBI even after they had received treatment. This suggests that there is a high degree of neuroinflammation in the brain of such patients.

Microglia are resident macrophages in the brain that are not only extremely active in injury and disease but also play a key role in maintaining brain health and function. Genetic damage to microglia may result in neurodegenerative diseases caused by triggering receptor expressed on myeloid cells [28–30]. The discovery of unique microglial subsets in some diseases can help to increase understanding of the function of microglia [31]. In the current study, LPS-treated microglia showed high levels of expression of multiple miRNAs, such as hsa-miR-193b-3p and hsa-miR-122-5p, which were also highly expressed in the peripheral blood of patients with TBI. This suggests that abnormally high levels of expression of miRNAs in the peripheral blood of patients with TBI may be associated with immune activation of microglia. In addition, we found that hsa-miR-193b-3p and hsa-miR-122-5p were associated with the severity of clinical consciousness disorders in patients with TBI. This is significant, as although many studies have explored the potential roles of miRNAs in the pathological progression of TBI in animal models, there remains a lack of high-quality retrospective studies on the clinical utility of TBI-associated miRNAs. Furthermore, ROC curve analysis showed that hsa-miR-122-5p and hsa-miR-193b-3p could possibly serve as biomarkers of TBI persisting after treatment. Long-term follow-up of patients with TBI and continual determination of the changes in the expression of these peripheral miRNAs in their blood will help to comprehensively evaluate the clinical utility of these miRNAs. Given that clinical trials for the treatment of TBI have not yielded satisfactory results [25, 32], exploration of the roles played by miRNAs in the mechanisms of the immune response of patients with TBI may result in the development of new strategies for the treatment of TBI.

## Conclusion

Our results showed significant increases in the levels of expression of certain miRNAs in the peripheral blood of patients with TBI after they had received treatment compared with before they had received treatment. We also found that this was highly likely to be due to microglial activation and was correlated with the severity of TBI. These findings could serve as important references to support the development of new therapeutic strategies for TBI based on manipulating the function of microglia.

### Supplementary Information

**Additional file 1: Fig. S1.** Identification of the number of miRNAs in each peripheral blood sample from patients with TBI before and after they had received treatment, respectively.

**Additional file 2: Fig. S2.** GO enrichment analysis (A) Bar chart of significance of target gene enrichment pathways in the GO database.

**Additional file 3: Fig. S2.** GO enrichment analysis. (B) Bar chart of target gene enrichment pathways in the GO database.

**Additional file 4: Fig. S3.** KEGG enrichment analysis. (A) Histogram of significance of target gene enrichment pathways in the KEGG. The pathways are indicated on the vertical axis, and the percentages of the number of pathways to all miRNA target genes are indicated on the horizontal axis. The darker the colour of a bar, the smaller the *Q*-value it represents; and the numerical value on a column represents the number and *Q*-value of the corresponding pathway.

**Additional file 5: Fig. S3.** KEGG enrichment analysis. (B) Bar chart of target gene enrichment pathways in the KEGG. The pathways are indicated on the vertical axis, and the percentages of the number of pathways to all miRNA target genes are indicated on the horizontal axis. The darker the colour of a bar, the smaller the *Q*-value it represents; and the numerical value on a column represents the number and *Q*-value of the corresponding pathway.

**Additional file 6: Table S1.** Expression profiles of miRNAs in peripheral blood of patients with TBI before and after treatment.

**Additional file 7: Table S2.** Detailed information of ROC curves for miRNAs in the peripheral blood of TBI patients.

## Data Availability

The datasets produced and/or analysed are available from the corresponding author upon reasonable request.
