# Supplementary figures and images for: Abnormal levels of expression of microRNAs in peripheral blood of patients with traumatic brain injury are induced by microglial activation and correlated with severity of injury
Source: Eur J Med Res. 2024 Mar 20;29:188. doi: 10.1186/s40001-024-01790-y (PMC10953077; doi:10.1186/s40001-024-01790-y)

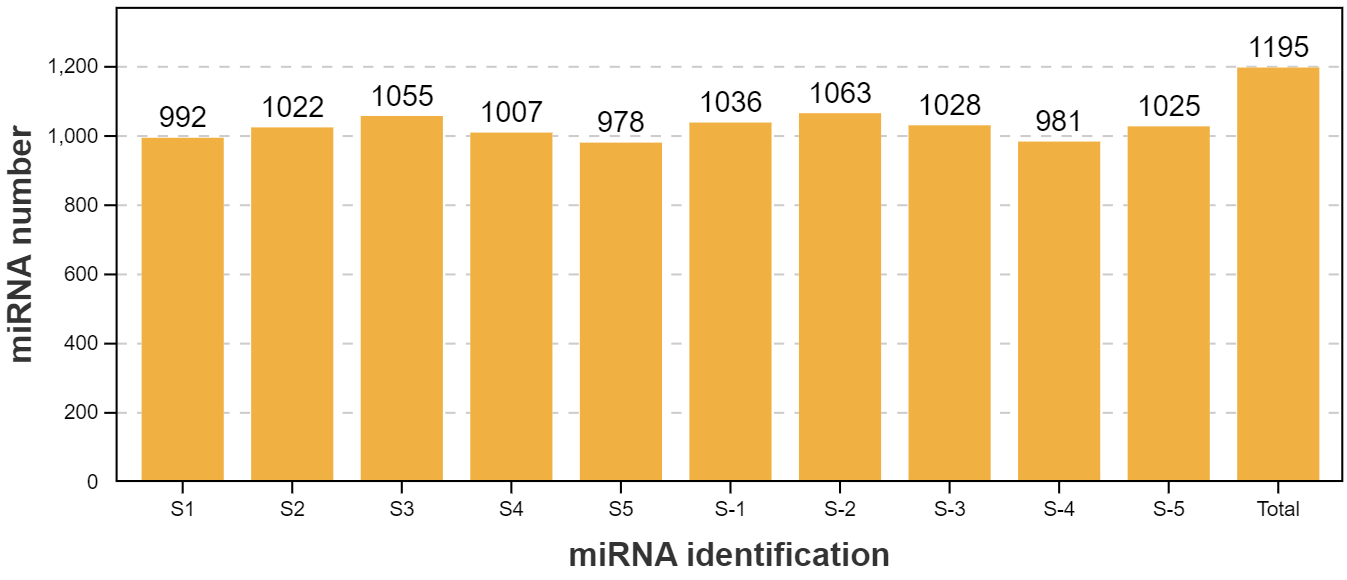

Supplement: Supplementary file 1 — Additional file 1: Fig. S1. Identification of the number of miRNAs in each peripheral blood sample from patients with TBI before and after they had received treatment, respectively. [file 40001_2024_1790_MOESM1_ESM.jpeg]

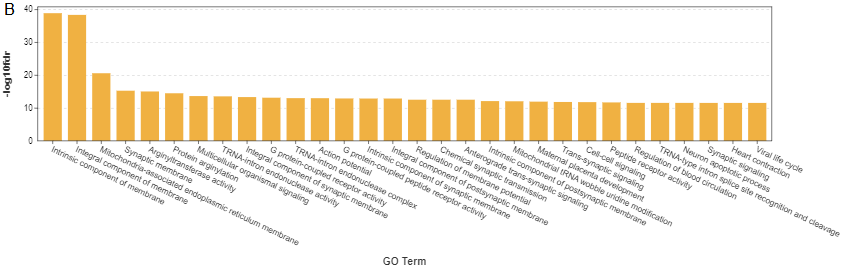

Supplement: Supplementary file 2 — Additional file 2: Fig. S2. GO enrichment analysis (A) Bar chart of significance of target gene enrichment pathways in the GO database. [file 40001_2024_1790_MOESM2_ESM.jpeg]

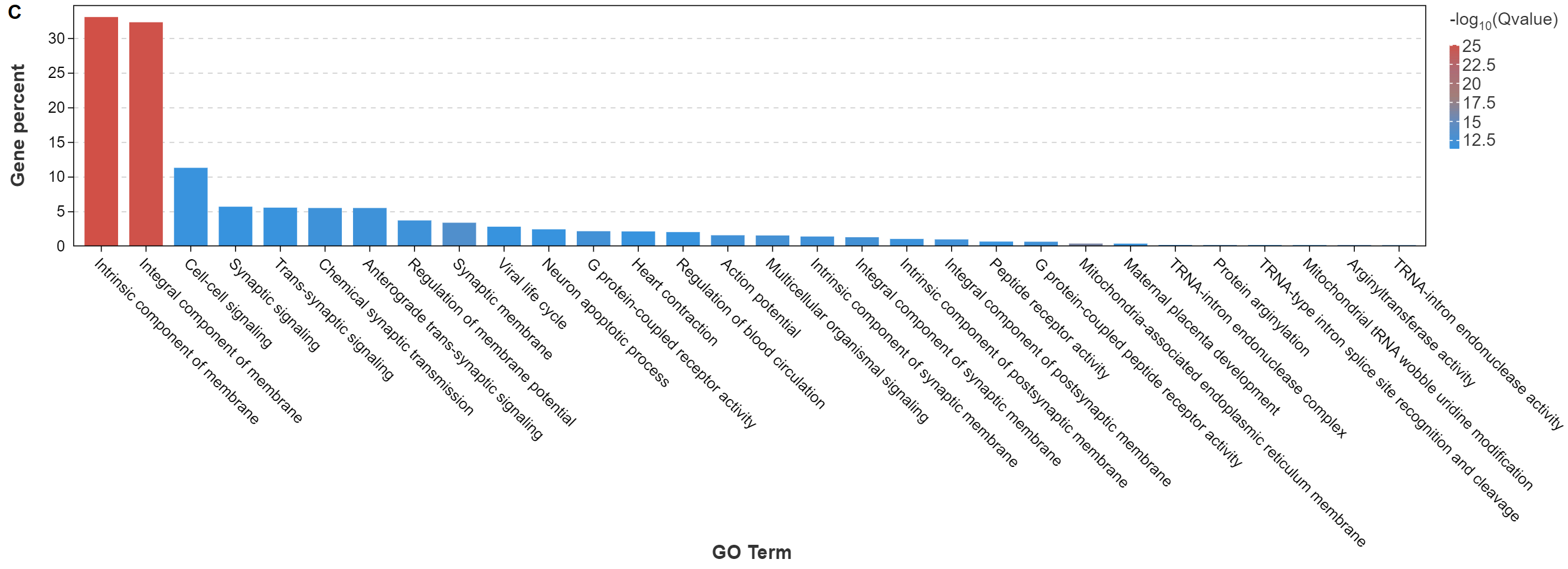

Supplement: Supplementary file 3 — Additional file 3: Fig. S2. GO enrichment analysis. (B) Bar chart of target gene enrichment pathways in the GO database. [file 40001_2024_1790_MOESM3_ESM.jpeg]

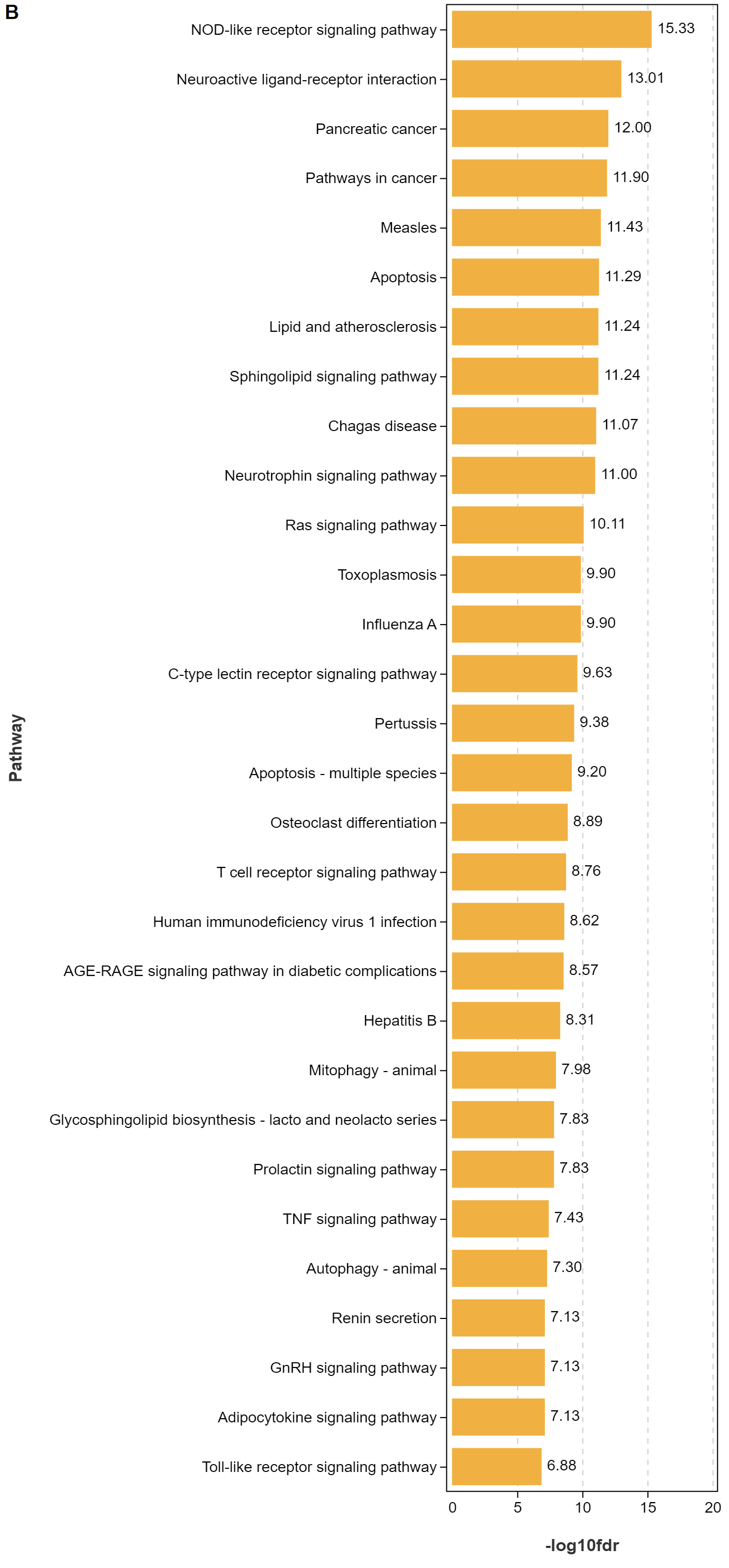

Supplement: Supplementary file 4 — Additional file 4: Fig. S3. KEGG enrichment analysis. (A) Histogram of significance of target gene enrichment pathways in the KEGG. The pathways are indicated on the vertical axis, and the percentages of the number of pathways to all miRNA target genes are indicated on the horizontal axis. The darker the colour of a bar, the smaller the Q-value it represents; and the numerical value on a column represents the number and Q-value of the corresponding pathway. [file 40001_2024_1790_MOESM4_ESM.jpeg]

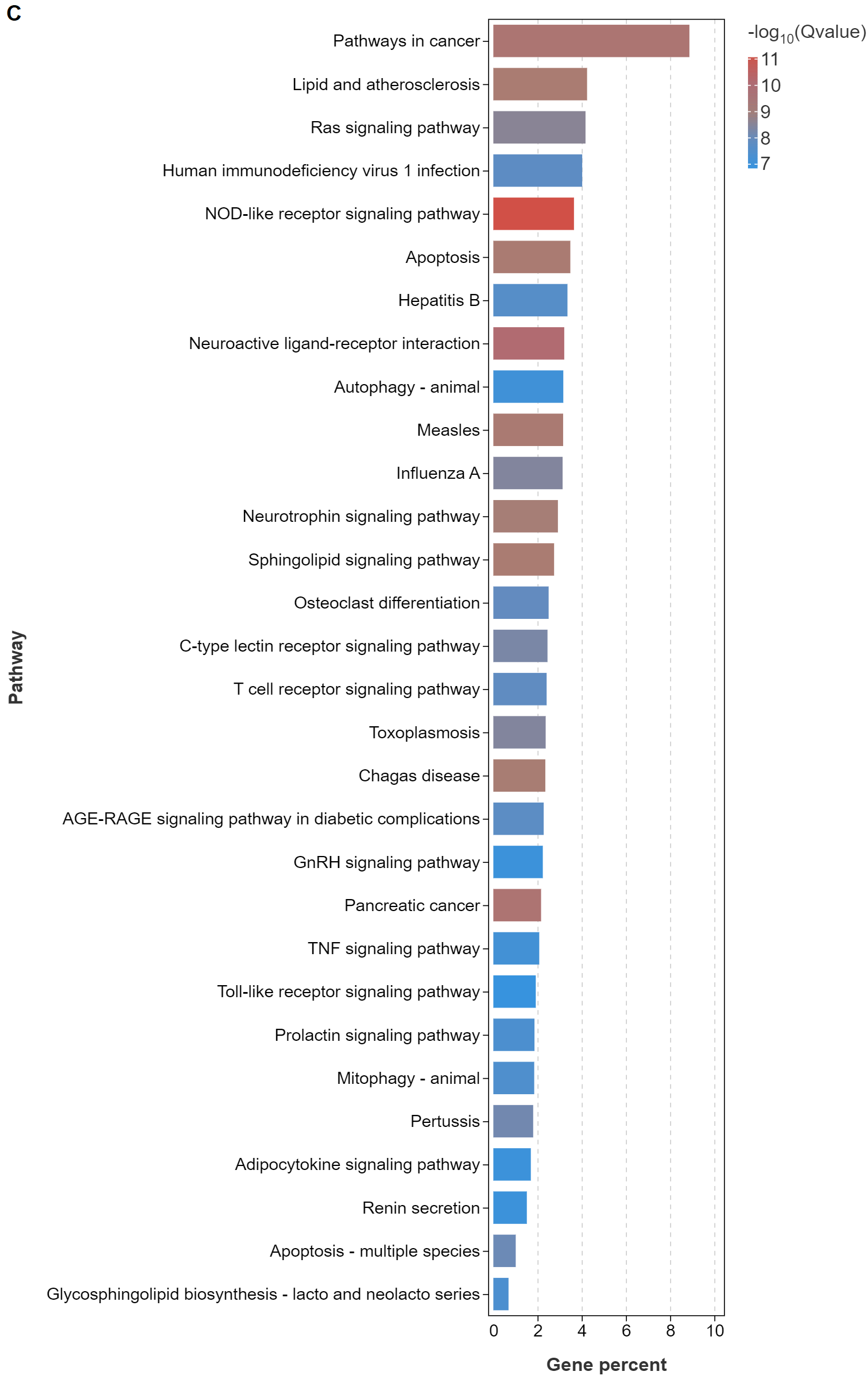

Supplement: Supplementary file 5 — Additional file 5: Fig. S3. KEGG enrichment analysis. (B) Bar chart of target gene enrichment pathways in the KEGG. The pathways are indicated on the vertical axis, and the percentages of the number of pathways to all miRNA target genes are indicated on the horizontal axis. The darker the colour of a bar, the smaller the Q-value it represents; and the numerical value on a column represents the number and Q-value of the corresponding pathway. [file 40001_2024_1790_MOESM5_ESM.jpeg]
