# Supplementary material for: Abnormal levels of expression of microRNAs in peripheral blood of patients with traumatic brain injury are induced by microglial activation and correlated with severity of injury
Source: Eur J Med Res. 2024 Mar 20;29:188. doi: 10.1186/s40001-024-01790-y (PMC10953077; doi:10.1186/s40001-024-01790-y)
Supplement: Supplementary file 7 — Additional file 7: Table S2. Detailed information of ROC curves for miRNAs in the peripheral blood of TBI patients. [file 40001_2024_1790_MOESM7_ESM.docx]

| **Table S2.** Detailed information of ROC curves for miRNAs in the peripheral blood of TBI patients. | | | | | | |
| --- | --- | --- | --- | --- | --- | --- |
| Items | AUC | 95% CI | Cut off value | Sensitivity | Specificity | *P* - value |
| hsa-miR-122-5p | 0.933 | 88.84%-97.84% | 1.375 | 0.76 | 0.98 | *** |
| hsa-miR-193b-3p | 0.864 | 79.43%-93.41% | 1.115 | 0.78 | 0.80 | *** |
